# Report of Prof. Gross on Malignant Diseases

**Published:** 1853-08

**Authors:** 


*Report of Prof. Gross on Malignant diseases.*—The following abstract was read at the last meeting of the American Medical Association. Prof. Gross' report in full will appear in the Transactions of the Association:

From the facts and statements which have now been presented, embracing the opinions of many of the most intelligent, experienced and distinguished practitioners in different ages, and in different parts of the world, the following conclusions may be legitimately deduced:

*First*—That cancerous affections, particularly those of the mammary gland, have always, with a few rare exceptions, been regarded by practitioners as incurable by the knife and escharotics. This opinion, commencing with Hippocrates, the father of medicine, has prevailed from the earliest records of the profession, to the present moment. Nature never cures a disease of the kind; nor can this be effected by any medicine, or internal remedies known to the profession.

*Secondly*—That excision, however early and thoroughly executed, is nearly always, in genuine cancer, followed by relapse, at a period varying from a few weeks to several months, from the operation.

*Thirdly*—That nearly all practitioners, from the time of Hippocrates to the present day, have been, and are still averse to any operation for the removal of cancerous tumors, after the establishment of ulceration, rapid growth, firm adhesion, organic change in the skin, lymphatic invasion, the cancerous dyscracy, or serious constitutional derangements; on the ground that, if had recourse to, under these circumstances, the malady almost invariably recurs in a very short time, and frequently destroys the patient more rapidly than when it is permitted to pursue its own course.

*Fourthly*—That in all cases of *acute* carcinoma, or, in other words, in all cases of this disease, attended with very rapid development and great bulk of the tumor, extirpation is improper and unjustifiable, inasmuch as it will only tend to expedite the fatal result, which, under such circumstances, always takes place in a very short time.

*Fifthly*—That all operations performed for the removal of encephaloid cancer and its different varieties, are more certainly followed by rapid relapse than operations performed upon schirrus or hard cancer.

*Sixthly*—That in nearly all the operations for cancerous diseases, hitherto reported, the history has been imperfectly presented, being deficient in the details which are necessary to a complete and thorough understanding of the subject in each case. This remark is particularly true in reference to the diagnosis of the malady, the minute examination of the morbid structure, and the history of the case after the operation, as to the period of relapse, the time and nature of the patient's death, and the result of the post-mortem examination.

*Seventhly*—That cancerous affections of the lip and skin, now usually described under the name of cancroid diseases, are less liable to relapse after extirpation than genuine cancerous maladies, or those which are characterized by the existence of the cancer cell and cancer juice.

*Eighthly*—That, although practitioners have always been aware, from the earliest professional records, of the great liability of cancer to relapse after extirpation, a great majority of them have always been, and still are, in favor of operation in the early stage of the disease, especially in schirrus, before the tumor has made much progress, or before there is any disease of the lymphatic ganglions, or evidence of the cancerous cachexy.

*Ninthly*—That many cases of tumors, especially tumors of the breast and testicle, supposed to be cancerous, are in reality not cancerous, but of a benign character, and consequently, readily curable by ablation, whether effected by the knife or by escharotics. It is to this circumstance that we must ascribe the astonishing success which is said to have attended the practice of Hill, of Scotland, Nooth, of England, and Flajani, of Italy.

*Tenthly*—That all operations insist upon the most thorough excision possible; removing not merely the diseased mass, but also a portion of the surrounding and apparently healthy tissues, as well as the enlarged and indurated ganglions.

*Eleventhly*—That the practice has always prevailed, and still obtains, to save, if possible, a sufficient amount of healthy integument to cover the wound, and if possible, to unite it, by the first intention; on the ground that these precautions will tend much to retard, if not to prevent, a recurrence of the disease.

*Twelfthly*—That much stress is laid by writers upon a properly regulated diet, and attention to the bowels and secretions after operation, as means of retarding and preventing relapse.

*Thirteenthly*—That there is no remedy, medicine or method of treatment which has the power, so far as we are enabled to judge of its virtues, of preventing the reproduction of the morbid action after operation, no matter how thoroughly it may be performed.

*Fourteenthly*—That life has occasionally been prolonged and even saved by operation after relapse, as in some of the remarkable cases mentioned in a previous part of this report; but that, as a general rule, such a procedure is as incompetent to effect a permanent cure as a first extirpation.

